# The prevalence and risk factors for tuberculosis among healthcare workers in Yogyakarta, Indonesia

**DOI:** 10.1371/journal.pone.0279215

**Published:** 2023-05-18

**Authors:** Stephanie Main, Rina Triasih, Jane Greig, Arif Hidayat, Immanuel Billy Brilliandi, Syarifah Khodijah, Geoff Chan, Nova Wilks, Amy Elizabeth Parry, Betty Nababan, Philipp du Cros, Bintari Dwihardiani

**Affiliations:** 1 Tuberculosis Elimination and Implementation Science Working Group, Burnet Institute, Melbourne, Victoria, Australia; 2 National Centre for Epidemiology and Population Health, Australian National University, Canberra, Australian Capital Territory, Australia; 3 Centre of Tropical Medicine, Faculty of Medicine, Public Health and Nursing, Universitas Gadjah Mada, Yogyakarta City, Yogyakarta, Indonesia; 4 Department of Paediatric, Faculty of Medicine, Public Health and Nursing, Universitas Gadjah Mada/Dr. Sardjito Hospital, Yogyakarta City, Yogyakarta, Indonesia; University of Cape Town, UNITED STATES

## Abstract

Healthcare workers (HCWs) are at risk of contracting TB, particularly when in high tuberculosis (TB) burden settings. Routine surveillance data and evidence are limited on the burden of TB amongst HCWs in Indonesia. We aimed to measure the prevalence of TB infection (TBI) and disease among HCWs in four healthcare facilities in Yogyakarta province in Indonesia, and explore risk factors for TBI. A cross-sectional TB screening study targeted all HCWs from four pre-selected facilities (1 hospital, 3 primary care) in Yogyakarta, Indonesia. Voluntary screening included symptom assessment, Chest X-ray (CXR), Xpert MTB/RIF (if indicated) and tuberculin skin test (TST). Analyses were descriptive and included multivariable logistic regression. Of 792 HCWs, 681 consented (86%) to the screening; 59% (n = 401) were female, 62% were medical staff (n = 421), 77% worked in the one participating hospital (n = 524), and the median time working in the health sector was 13 years (IQR: 6–25 years). Nearly half had provided services for people with TB (46%, n = 316) and 9% reported ever having TB (n = 60). Among participants with presumptive TB (15%, n = 99/662), none were diagnosed microbiologically or clinically with active TB disease. TBI was detected in 25% (95% CI: 22–30; n = 112/441) of eligible HCWs with a TST result. A significant association was found between TB infection and being male (adjusted Odds Ratio (aOR) 2.02 (95%CI: 1.29–3.17)), currently working in the participating hospital compared to primary care (aOR 3.15 (95%CI: 1.75–5.66)), and older age (1.05 OR increase per year of life between 19–73 years (95%CI: 1.02–1.06)). This study supports prioritisation of HCWs as a high-risk group for TB infection and disease, and the need for comprehensive prevention and control programs in Indonesia. Further, it identifies characteristics of HCWs in Yogyakarta at higher risk of TBI, who could be prioritised in screening programs if universal coverage of prevention and control measures cannot be achieved.

## Introduction

Tuberculosis (TB) remains one of the most burdensome and deadliest infectious diseases worldwide, causing an estimated 10.6 million cases and 1.6 million deaths in 2021 [1]. Low- and middle-income countries (LMICs) bear the brunt of TB’s impact, with more than 95% of deaths occurring in these countries. Eight LMICs account for two thirds of the global caseload, one of which is Indonesia [1].

Healthcare workers (HCWs) are at an increased risk of contracting TB compared to the general population [2–4]. The burden of TB amongst HCWs is higher in LMICs, although estimates vary considerably. A systematic review published in 2019 found TBI prevalence by positive tuberculin skin test (TST) ranged from 14–98% (mean 49%) among HCWs in LMICs, with higher prevalence among HCW populations in higher TB burden settings (defined as ≥300 cases per 100,000 population) [5]. Earlier reviews reported similar variability with annual TBI incidence among HCWs ranging between 0.5–14.3% [6, 7]. The reason for this increased burden amongst HCWs in LMICs is multifaceted. TB infection (TBI) risk is higher in LMICs where persistent exposure to infected individuals is more likely, and infection prevention and control practices might be insufficient or inconsistent [6]. Further, various occupational risk factors such as occupation type and contact with TB patients play a role in increasing infection risk, although evidence for risks are varied across contexts [5–7].

Meeting ambitious End TB strategy targets by 2035 requires a well-resourced, skilled and healthy healthcare workforce [8]. The burden of TB in HCWs highlights the negative impact occupational TB and nosocomial TB transmission could have on meeting End TB targets and wider TB elimination. The burden also highlights the need for further research, and targeted policy and programming among this high priority population.

Indonesia has the third highest TB caseload internationally and an annual disease incidence of 301 per 100,000 [9]. Surveillance data on the burden of TB among HCWs in Indonesia are limited, with estimates of reported TBI burden ranging from 24% among HCWs in primary healthcare centres (PHCs) to 77% in tertiary hospitals [10, 11]. There is limited information on TB burden in HCWs across many provinces including Yogyakarta. We aimed to measure the prevalence of infection and disease among HCWs in four healthcare facilities in Yogyakarta and explore risk factors for infection.

## Materials and methods

### Study setting

Yogyakarta province, located in central southern Java, has an estimated TB disease incidence rate between 250–300 per 100,000 population and an estimated case detection rate of 34.2% [12]. The Yogyakarta health system, like Indonesia’s, encompasses several jurisdictional levels and includes both public and private providers [13]. District level public and private hospitals along with PHCs provide TB services comprising case detection, diagnosis, treatment and prevention [13]. Patients can attend PHCs for care, receive a referral to private or public hospitals or can attend non-referral-based outpatient services at the hospitals. The 2016–17 Indonesian TB Inventory Study estimated that 37% of TB patients in Indonesia sought care at a private hospital.

### Study design, site selection and study population

The study selected all healthcare facilities delivering TB care within two sub-districts of two districts of Yogyakarta province. Selection of healthcare facilities in this study was targeted, based on the respective sub-district’s involvement in a TB Reach funded active case finding program [14]. Health office consultation, low case detection rates, geography and operational challenges such as near absence of contact tracing and testing, were reasons for sub-district selection into the program. The healthcare facilities selected consisted of one private hospital treating between 100–120 TB patients per year (2018–2019) and three public PHCs treating 4–20 TB patients per year (2018–2019).

The target population for this cross-sectional study was all healthcare facility staff from the four pre-selected facilities aged ≥17 years (legal age of adulthood in Indonesia), including non-medical (e.g., management and support services) and medical staff. There were 792 HCWs across the four facilities; 613 at the private hospital and 179 staff across the PHCs. No sampling methodology was used in this study as we invited the entire target population to participate.

### Participant recruitment

Information about the screening, including its purpose, date, and location were disseminated through advertisements in staff break rooms, online notification through the facility’s human resource network, and awareness-raising sessions for staff before the period of screening. The sessions were conducted by the study team and included information on TB, TB burden in HCWs, the value of screening, the screening process, dates and location of the screening, the consent process and participant confidentiality. The study was voluntary, free of charge, and participants did not receive incentives for participating.

### Data collection

Screening of health workers was conducted for one week at each PHC and two weeks at the private hospital between December 2020 to May 2021. HCWs were invited to join the study at a time that was convenient to them, which could be prior to, during or after work hours. Trained research staff screened and interviewed the participants.

TB infection and disease screening included symptom assessment, Chest X-ray (CXR), one spot sputum sample for Xpert MTB/RIF testing (if indicated) and TST. Culture was used when there were indeterminate or positive Xpert MTB/RIF results. TBI was classified as TST positivity (>10mm induration or >5mm in immunosuppressed participants) with no evidence of clinically manifest active TB [15, 16]. The study utilised the Indonesian National TB Program (NTP) laboratories, tests and supplies. The TB testing algorithm balanced sensitivity, specificity, timeliness, capacity of laboratories and burden on routine testing and aligned with NTP guidelines. Standard COVID-19 infection prevention and control procedures and strategies were implemented throughout the study, with participants screening positive for COVID-19 symptoms offered a PCR test. People who tested positive for COVID-19 and/or those who had recently received a COVID-19 vaccine were followed up for TB screening during additional screening days. All tests were performed by trained healthcare professionals.

Final TB diagnoses were made by the study team doctor, based on Indonesian and WHO definitions/criteria. Diagnostic results and information on treatment options were disseminated by formal letter if the participant screening outcome was negative, or through a doctor consultation if TBI was diagnosed. Pending consent, participant eligibility to take TB preventive treatment (TPT) was assessed, and when relevant, participants were supported to attend their chosen healthcare facility to initiate TPT according to the National guidelines.

Interview and examination data were entered electronically at the point of care using REDCap [17]. The study interview collected data on participant demographic, clinical and occupational information including age, sex, healthcare role, time working in the health sector, history of TB, comorbidities and other risk factors. All information was voluntary to disclose.

### Data analysis

Re-identifiable study data were exported into Stata v15 (StataCorp, College Station, TX, USA).

Descriptive analyses were utilised to measure TBI prevalence and examine cohort characteristics. Categorical variables were reported as numbers and proportions. Continuous variables were assessed for normality and reported as median and interquartile range (IQR).

Risk factors associated with TBI were investigated by examining demographic, occupational, and clinical factors using univariable and multivariable logistic regression. The effect was expressed as an unadjusted and adjusted odds ratio (OR) with 95% confidence intervals (95% CI). Variables were added to the multivariable model if p < 0.2 in the univariable analyses. Those recorded only for sub-sets of the sample or demonstrating collinearity were excluded. Backward selection was employed, and selection stopped when the multivariable model Likelihood Ratio test showed only a statistically non-significant improvement in modelling. Levels of significance in the multivariable model and statistical tests were set at 5%.

### Ethical approval

Ethical approval was obtained from the Institutional Ethical Review Board of the Faculty of Medicine, Public Health and Nursing, University of Gadjah Mada, Indonesia (number KE/FK/0323/EC) and the Alfred Hospital Human Research Ethics Committee, Australia (number 639–20). All participants provided informed written consent.

## Results

Participation in the study was high with 86% (n = 681/792) of registered HCWs at the facilities participating, ranging from 73–94% by facility. Of the 681 participants the median age was 40 years (IQR:30–48), 59% (n = 401) were female, 71% (n = 486) reported completing a university degree, and participant’s median household size was 4 people (IQR:2–5 people) (Table 1). Nearly two thirds of participants identified as medical staff (62%, n = 421), mainly nurses (n = 250, 37%), 77% worked in the hospital (n = 524), and the median time working in the health sector was 13 years (IQR: 6–25 years) (Table 1). Nearly half had provided support services or medical care for people with TB (46%, n = 316) and 36% (n = 243) had received formal or informal (e.g., information sessions) TB training outside of their professional qualification.

**Table 1:** Demographic, occupational and clinical characteristics of healthcare workers screened for TB in primary health facilities and a private hospital in two sub-districts of Yogyakarta Indonesia.

Characteristics	Total (n,%)
**Total**	**681**
*Demographic Characteristics*	
**Age** median [IQR]	40 [30–48]
**Sex**	
Male	280 (41.1)
Female	401 (58.9)
**Education**	
High School Degree or Less	195 (28.6)
University Degree	486 (71.4)
**Household size**, median [IQR]	4 [3–5]*
**Household expenses**	
< Rp. 2.2 million per month (low)	188 (27.6)
Rp. 2.2–5 million per month (medium)	364 (53.5)
> Rp. 5 million per month (high)	105 (15.4)
Missing	24 (3.5)
*Occupational Characteristics*	
**Health facility type**	
Hospital	524 (76.9)
Primary Health Centre	157 (23.1)
**Occupation category**	
*Medical*	*421 (61*.*8)*
Nurse	250 (37.0)
Dentist	2 (0.3)
Laboratory Scientists	18 (2.6)
Nutritionist	15 (2.2)
Environmental Health	2 (0.3)
Epidemiologist	6 (0.9)
Radiographer	9 (1.3)
Midwife	47 (6.9)
Rehabilitation staff (e.g. physiotherapist)	14 (2.1)
Health Promotion Nurse	2 (0.3)
Pharmacist and assistants	43 (6.3)
Doctor	12 (1.8)
Psychologist	1 (0.1)
*Non-Medical*	*258 (37*.*9)*
Administrative staff	82 (12.0)
Management	4 (0.6)
Community Health Workers	23 (3.4)
Other non-medical personnel (e.g. cleaners and cooks)	149 (21.9)
*Missing*	*2 (0*.*3)*
**Length of time working in the health sector (years)**, median [IQR]	13 [6–25]
**Length of time at this health facility (years)**, median [IQR]	11 [4–24]^
**Provided services / care to a person diagnosed with tuberculosis**	
No	365 (53.6)
Yes	316 (46.4)
***Length of time provided services / care to people with TB (years)***, median [IQR]	7 [2–17]
**Received TB training outside of professional qualification**	
No	436 (64.0)
Yes	243 (35.7)
Missing	2 (0.3)
**Received training on personal protective equipment (non-disease specific)**	
No	114 (16.7)
Yes	565 (83.0)
Missing	2 (0.3)
*Clinical characteristics*	
**Nutritional status**^#^	
Underweight (BMI < 18.5)	41 (6.0)
Normal weight (BMI > = 18.5 to < 23)	190 (27.9)
Overweight (BMI > = 23 to < 25)	126 (18.5)
Obese (BMI > = 25)	324 (47.6)
**Smoking status**	
No, never smoked	488 (71.7)
No, but previously smoked	94 (13.8)
Currently smoke	99 (14.5)
**Self-reported diabetes mellitus status**	
No	617 (90.6)
Yes	24 (3.5)
Unsure	40 (5.9)
**Self-reported HIV Status**	
Positive	0 (0)
Negative	190 (27.9)
Unsure	491 (72.1)
**Self-reported Pregnancy Status Among Females**	
No	390 (97.3)
Yes	11 (2.7)
**Lived with someone who had TB for at least one day**	
No	582 (85.5%)
Yes	91 (13.4%)
Unsure	8 (1.2%)
***Timing of living with someone with TB***	
Before person with TB started treatment	79 (86.8)
After person with TB started treatment	11 (12.1)
Unsure	1 (1.1)
**Previously been treated for TB**	
No	621 (91.2)
Yes, finished treatment	59 (8.7)
Yes, currently on medication	1 (0.1)
*Treated*	

*656/681 participants responded

^ 678/681 participants responded

^#^ BMI classification for people born in Asia [18]

Among the 681 participants, 67% (n = 450) were overweight or obese, 29% currently (15%, n = 99) or previously (14%, n = 94) smoked, self-reported diabetes mellitus (DM) prevalence was 4% (n = 24), self-reported HIV status was known or disclosed among 28% (n = 190) of participants and all were negative [18] (Table 1). Ninety-one participants (13%, n = 91) reported ever having lived with someone who had TB, mostly (87%, n = 79/91) before the person started TB treatment. Sixty participants reported ever having TB (9%, n = 60), of whom 75% were females (n = 45), 73% (n = 44) were medical staff, the median time in the health sector was 10 years (IQR: 5–16 years), 60% (n = 36) had provided services for people with TB, and 17% (n = 10) had lived with someone with TB for at least one day (Table 2).

**Table 2:** Characteristics associated with self-reported previous TB disease among healthcare workers in primary health facilities and a private hospital in two sub-districts of Yogyakarta Indonesia.

Characteristics	Total	No Previous TB History	History of TB Disease
	(n, %)	(n, %)	(n, %)
**Total**	**681**	**621**	**60**
*Demographic Characteristics*			
**Age (years)**, median [IQR]	40 (30.4–48.1)	40.8 (30.6–48.3)	33.7 (28.95–42.8)
**Sex**			
Female	401 (58.9)	356 (57.3)	45 (75.0)
Male	280 (41.1)	265 (42.7)	15 (25.0)
**Education**			
High School Degree or Less	195 (28.6)	185 (29.8)	10 (16.7)
University Degree	486 (71.4)	436 (70.2)	50 (83.3)
**Household Size (people)**, median [IQR]	4 (3–5)	4 (3–5)	4 (3–5)
**Household expenses** *			
< Rp. 2.2 million per month (low)	188 (28.6)	176 (29.3)	12 (21.1)
Rp. 2.2–5 million per month (medium)	364 (55.4)	330 (55.0)	34 (59.6)
> Rp. 5 million per month (high)	105 (16.0)	94 (15.7)	11 (19.3)
*Occupational Characteristics*			
**Health Facility**			
Primary Health Facility	157 (23.1)	144 (23.2)	13 (21.7)
Hospital	524 (76.9)	477 (76.8)	47 (78.3)
**Occupation**			
Non-Medical	258 (37.9)	242 (39.0)	16 (26.7)
Medical	421 (61.8)	377 (60.7)	44 (73.3)
Missing	2 (0.3)	2 (0.3)	0 (0)
**Length of time working in the health sector (years)**, median [IQR]	13 (6–25)	14 (6–25)	10 (5–16)
**Length of time in working in this facility (years)**, median [IQR]	11 (4–24)	12 (4–24)	9.5 (3–15)
**Provided services / care to a person diagnosed with tuberculosis**			
No	365 (53.6)	341 (54.9)	24 (40.0)
Yes	316 (46.4)	280 (45.1)	36 (60.0)
***Length of time provided services / care to people with TB (years)***, median [IQR]	7 (2–17)	7 (2–17)	10 (4–20)
**Received TB training outside of professional qualification** ^			
No	436 (64.0)	398 (64.1)	38 (63.3)
Yes	243 (35.7)	221 (35.6)	22 (36.7)
Missing	2 (0.3)	2 (0.3)	0 (0)
**Received training on personal protective equipment (non-disease specific)**			
No	114 (16.7)	105 (16.9)	9 (15.0)
Yes	565 (83.0)	514 (82.8)	51 (85.0)
Missing	2 (0.3)	2 (0.3)	0 (0)
*Clinical characteristics*			
**Nutritional status**			
Underweight (BMI < 18.5)	41 (6.0)	39 (6.3)	2 (3.3)
Normal weight (BMI > = 18.5 to < 23)	190 (27.9)	165 (26.6)	25 (41.7)
Overweight (BMI > = 23 to < 25)	126 (18.5)	113 (18.2)	13 (21.7)
Obese (BMI > = 25)	324 (47.6)	304 (49.0)	20 (33.3)
**Smoking Status**			
No—never smoked	488 (71.7)	441 (71.0)	47 (78.3)
No—but previously smoked	94 (13.8)	89 (14.3)	5 (8.3)
Yes	99 (14.5)	91 (14.7)	8 (13.3)
**Self-Reported Diabetes Mellitus Status**			
No	617 (90.6)	561 (90.3)	56 (93.3)
Yes	24 (3.5)	23 (3.7)	1 (1.7)
Unsure	40 (5.9)	37 (6.0)	3 (5.0)
**Self-Reported HIV Status**			
Positive	0 (0)	0 (0)	0 (0)
Negative	190 (27.9)	167 (26.9)	23 (38.3)
Unsure	491 (72.1)	454 (73.1)	37 (61.7)
**Self-Reported Pregnancy Status Among Females**			
No	390 (97.3)	345 (96.9)	45 (100.0)
Yes	11 (2.7)	11 (3.1)	0 (0)
**Lived with someone who had TB for at least one day**			
No	582 (85.5)	533 (85.8)	49 (81.7)
Yes	91 (13.4)	81 (13.0)	10 (16.7)
Unsure	8 (1.2)	7 (1.1)	1 (1.7)

*657/681 participants responded

^ 678/681 participants responded

During screening, 1% (n = 5/681) of participants reported cough for more than two weeks or haemoptysis. Of those CXR screened (n = 662; eight participants refused and 11 were pregnant), 36% had a lung or pleural abnormality (n = 239/662), and 15% (n = 97/662) of findings were consistent with possible active TB disease requiring further investigation and assessment (Table 3). Of the 15% of participants (n = 99/662) with a combined symptom screen positive and/or a CXR suggestive of TB and who subsequently had further investigations including Xpert MTB/RIF testing and a clinical examination, none were diagnosed microbiologically (via Xpert MTB/RIF of sputum) or clinically with TB disease.

**Table 3:** Chest X-ray findings among healthcare workers with an abnormal chest X-ray in primary health facilities and a private hospital in two sub-districts of Yogyakarta Indonesia.

Chest X-Ray Abnormality	Suggestive of TB	Not Suggestive of TB	Total
**Total**	**N = 97** *	**N = 147** *	**N = 239** *
Infiltrates	93	0	93
Consolidation	0	0	0
Cavity	1	0	1
Calcification	1	1	2
Pleural Effusion	2	0	0
Fibrosis	5	11	16
Increased Bronchovascular Marking	46	102	148
Emphysematous Lung	1	3	4
Lymphadenopathy	1	1	2
Tented Diaphragm	1	1	2

*The denominator for the columns are the totals listed, not the total abnormalities listed. Some participants had more than one x-ray abnormality.

**Fig 1:**
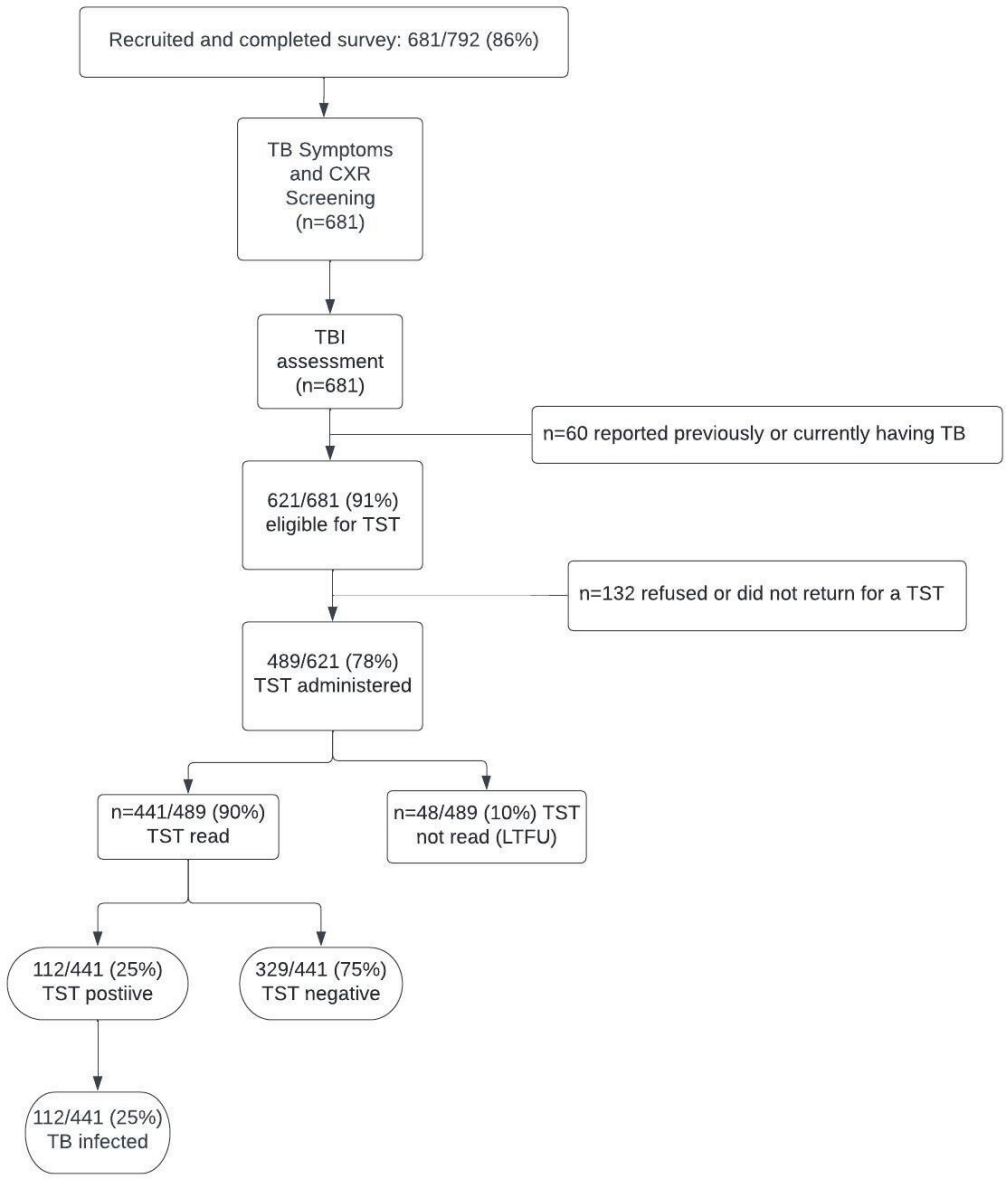
TB infection investigation flow among healthcare workers screened for TB in primary health facilities and a private hospital in two sub-districts of Yogyakarta Indonesia.

TST was offered to all participants who had not reported previously receiving TB treatment (n = 621). It was administered to 78% (n = 489/621) of those eligible and read in 90% (n = 441/489) of them (Fig 1). TBI was diagnosed in 25% (95% CI: 22–30) of HCWs (n = 112/441), most being HCWs in the hospital setting (86%; n = 96/112) (Table 3). TBI prevalence differed between the hospital setting (31%, 95% CI: 26–37%; n = 96/306) and PHCs (12%, 95% CI: 1–19%; n = 16/135).

Of the 112 participants diagnosed with TBI, 8% (n = 9) were underweight, 35% currently (19%, n = 21) or previously (16%, n = 18) smoked, 5% (n = 6) reported having DM and 80% (n = 90) were unsure of or unwilling to disclose their HIV status (Table 4). A significant association on multivariable logistic regression was found between TBI and being male (adjusted OR 2.02 (95%CI: 1.29–3.17)), currently working in the participating hospital compared to the PHCs (adjusted OR 3.15 (95%CI: 1.75–5.66)), and older age (1.05 OR increase per year of life between 19–73 years (95%CI: 1.02–1.06)) (Table 4).

**Table 4:** Characteristics associated with TB infection among healthcare workers in primary health facilities and a private hospital in two sub-districts of Yogyakarta Indonesia.

Characteristics	Total	No TB infection	TB infection	Unadjusted Odds Ratio (OR)	Adjusted Odds Ratio (aOR)
	(n, %)	(n, %)	(n, %)	[95% CI]	[95% CI]
**Total**	**441**	**329**	**112**		
**Age (years)**, median [IQR]	41.1 (31–48.8)	39.7 (30.5–48.1)	45.8 (36.3–51.9)	1.05 [1.02–1.07]	1.05 [1.02–1.06]
**Sex**					
Female	261 (59.2)	211 (64.1)	50 (44.6)	REF	REF
Male	180 (40.8)	118 (35.9)	62 (55.4)	2.22 [1.43–3.43]	2.02 [1.29–3.17]
**Education**					
High School Degree or Less	153 (34.7)	102 (31.0)	51 (45.5)	REF	-
University Degree	288 (65.3)	227 (69.0)	61 (54.5)	0.54 [0.35–0.83]	-
**Household Size (people)**, median [IQR]	4 (3–5)	4 (3–5)	4 (3–5)	1.12 [0.96–1.30]	-
**Household expenses**					
< Rp. 2.2 million per month (low)	129 (30.4)	94 (29.7)	35 (32.4)	REF	-
Rp. 2.2–5 million per month (medium)	231 (54.4)	178 (56.2)	53 (49.1)	0.80 [0.49–1.31]	-
> Rp. 5 million per month (high)	65 (15.3)	45 (14.2)	20 (18.5)	1.19 [0.62–2.30]	-
**Health Facility**					
Primary Health Facility	135 (30.6)	119 (36.2)	16 (14.3)	REF	REF
Hospital	306 (69.4)	210 (63.8)	96 (85.7)	3.40 [1.91–6.04]	3.15 [1.75–5.66]
**Occupation**					
Non-Medical	198 (44.0)	141 (42.9)	57 (50.9)	REF	-
Medical	242 (55.0)	187 (56.8)	55 (49.1)	0.72 [0.47–1.12]	-
**Length of time working in the health sector (years)**, median [IQR]	15 (7–25)	13 (7–24.3)	22.5 (8–29)	1.03 [1.01–1.06]	-
**Length of time in working in this facility (years)**, median [IQR]	12 (5–24)	11 (5–24)	20 (5.5–27.5)	1.04 [1.01–1.06]	-
**Provided services / care to a person diagnosed with tuberculosis**					
No	272 (61.7)	208 (63.2)	64 (57.1)	REF	-
Yes	169 (38.3)	121 (36.8)	48 (42.9)	1.30 [0.83–1.99]	-
**Received TB training outside of professional qualification**					
No	294 (67.0)	215 (65.7)	79 (70.5)	REF	-
Yes	145 (33.0)	112 (34.3)	33 (29.5)	0.80 [0.50–1.28]	-
**Received training on personal protective equipment (non-disease specific)**					
No	82 (18.7)	62 (19.0)	20 (17.9)	REF	-
Yes	357 (81.3)	265 (81.0)	92 (82.1)	1.08 [0.62–1.88]	-
**Nutritional status**					
Underweight (BMI < 18.5)	32 (7.3)	23 (7.0)	9 (8.0)	REF	-
Normal weight (BMI > = 18.5 to < 23)	115 (26.1)	91 (27.7)	24 (21.4)	0.67[0.28–1.64]	-
Overweight (BMI > = 23 to < 25)	78 (17.7)	62 (18.8)	16 (14.3)	0.66 [0.26–1.70]	-
Obese (BMI > = 25)	216 (49.0)	153 (46.5)	63 (56.2)	1.05 [0.46–2.40]	-
**Smoking Status**					
No—never smoked	316 (71.7)	243 (73.9)	73 (65.2)	REF	-
No—but previously smoked	65 (14.7)	47 (14.3)	18 (16.1)	1.27 [0.70–2.33]	-
Yes	60 (13.6)	39 (11.9)	21 (18.8)	1.79 [0.99–3.24]	-
**Self-Reported Diabetes Mellitus Status**					
No	394 (89.3)	297 (90.3)	97 (86.6)	REF	
Yes	19 (4.3)	13 (4.0)	6 (5.4)	1.41 [0.52–3.82]	-
Unsure	28 (6.3)	19 (5.8)	9 (8.0)	1.45 [0.64–3.31]	-
**Self-Reported HIV Status**					
Positive	0 (0)	0 (0)	0 (0)	-	-
Negative	108 (24.5)	86 (26.1)	22 (19.6)	REF	-
Unsure	333 (75.5)	243 (73.9)	90 (80.4)	1.45 [0.85–2.45]	-
**Self-Reported Pregnancy Status Among Females**					
No	254 (97.3)	204 (96.7)	50 (100.0)	-	-
Yes	7 (2.7)	7 (3.3)	0 (0)	-	-
**Lived with someone who had TB for at least one day**					
No	376 (85.3)	286 (86.9)	90 (80.4)	REF	-
Yes	59 (13.4)	38 (11.6)	21 (18.8)	1.76 [0.98–3.15]	-
Unsure	6 (1.4)	5 (1.5)	1 (0.9)	0.64 [0.07–5.51]	-

## Discussion

In this study among HCWs in four healthcare facilities in Yogyakarta, no HCWs were diagnosed microbiologically or clinically with active TB disease, although 9% reported previous or current TB disease. TBI was diagnosed in 25% of those eligible for TST and who had the TST read; this was highest in staff from the hospital (31%, 95% CI: 26–37). A significant association was found between TBI and being male, currently working in the participating hospital and older age.

Annual incidence of active TB disease among HCWs has been reported to range from 69–5,780 per 100,000 population, and has consistently been documented to be higher than the general population [6]. No active disease was diagnosed during the study, which could suggest low disease incidence in this population of HCWs, adequate disease diagnosis and detection programs within these health facilities, or could be due to the cross-sectional nature of the screening. However, self-reported past TB disease prevalence of 9% suggests disease incidence may be relatively high. This prevalence was similar to reported past disease among HCWs in a tertiary hospital in Bandung, Indonesia [11].

Prevalence of TBI (using TST) among HCWs in LMICs has been reported to range from 14–98% (mean 49%), and is higher among HCWs in high burden settings (pooled estimate of 55%, 95% CI 41–69%) [5]. Our study findings of 25% were lower than this, and lower than a prevalence study conducted in a public tertiary hospital in Bandung, Indonesia that reported a 77% TST positivity [10, 11]. Our results, however, are similar to TBI prevalence studies across PHCs in Semarang, Indonesia (24%). Modelled community level TBI prevalence of 46% across Indonesia is also higher than the prevalence reported in our study [19]. However, Yogyakarta province does have a lower estimated TB incidence (between 250–300 per 100,000 population) compared to the Indonesian average, and infection may parallel this trend, although prevalence estimates for TBI do not exist at this jurisdictional level [12].

Being male and increasing age as non-occupational risk factors for TBI among HCWs are consistent with several international studies [5, 20–26]. Increased TB prevalence amongst men in LMICs is well documented [27, 28]. However the complex roles of biological, social, cultural and structural factors increasing this risk is still undetermined, as is the role of exposure risk behaviour [27]. Other studies have found other non-occupational factors associated with TBI such as education level, smoking status, diabetes mellitus and household contact with a person with TB [5, 6]. Our study did not find an independent association between these factors and TBI in this population of HCWs.

The evidence is varied surrounding occupational factors as risk factors for TBI, such as years of work in the healthcare sector, contact with a TB patient, occupation type or work location [5, 6, 20, 22, 23, 29]. Our study did not find any individual occupational risk factors associated with TBI in health services that provide TB services, although we did find a significantly higher odds of infection among those working in the participating private hospital compared to the primary care facilities. This may allude to differences in provision of care, especially TB care, health facility IPC policy and procedure, and health facility infrastructure and layout. Persistent occupational TB exposure and poor levels of TBI control implementation in LMICs is a known significant factor for the increased risk of HCWs contracting TB [30]. Higher risk of acquiring TB has been associated with certain work locations such as inpatient TB services, general medicine wards, emergency rooms and laboratories compared to medium risk for outpatient and surgical services, compared to the general populations [5]. Similarly, certain occupations have had higher incidence of infection such as allied health, physicians and nurses [5, 6].

Control measures, such as adoption of N95 respirators, engineering controls, staff screening, and FAST infection control strategies, may decrease annual TB incidence among HCWs by as much as 81% in high incidence settings [7]. Even administrative controls alone, such as routine patient and HCW screening, have proven effective in decreasing the risk for TBI among HCWs in LMICs, suggesting these should be prioritised [7]. The lack of implementation and scale-up of preventive measures, particularly failure to rapidly diagnose and treat TB, are primarily responsible for nosocomial transmission [30]. The World Health Organization’s policy on TBI control in healthcare facilities recommends conducting routine screening and surveillance of TB disease among HCWs [4]. Despite the benefits and recommendations, the implementation of these policies, IPC procedures and surveillance in high burden and/or LMICs is low [30]. During the study period, healthcare facilities in Indonesia did not routinely screen for active TB or TBI or provide preventive therapy for their staff, even though HCWs have been listed as high-risk in the 2016–2020 National Strategic Plan and should be provided with these services [31]. Budget distribution, the lack of a national guideline for HCW screening and lack of healthcare facility awareness have been listed as reasons for this lack of implementation.

This study has some limitations. Primarily, the cross-sectional design and target population of all staff at pre-selected study sites meant that while all facilities in the sub-districts participated, the prevalence of TB among HCWs and characteristics associated with TBI may not be generalisable to the district, provincial or sub-national level. Ascertaining the acquisition source for infection was not possible, with community sources possible in addition to healthcare facility sources. Secondly, 14% of HCW registered at the facilities did not participate, close to 20% of eligible participants did not have a TST administered, and 10% were loss to follow up for TST reading. Attrition at each stage may have introduced bias and potentially over or under-estimated the prevalence and association estimates. The main reason for not having a TST administered was due to the recommendation to delay TST for 4-weeks post COVID-19 vaccination [32]. The screening service did return after the vaccination roll-out at the facility, however, few participants returned. Thirdly, the limitations with TST’s sensitivity and specificity may have over or underestimated the prevalence of TBI [33]. Fourthly, categorising HCW occupation, due to small numbers, may have skewed the association with TBI. Finally, besides BMI and TB investigations, all clinical characteristics were self-reported, which may have introduced bias, over or under-estimating the association between characteristics and TBI, particularly for HIV. However, HCWs are not a high priority and risk population for HIV in Indonesia [34]. Strengths of this study include high participation proportions across the facilities, the use of radiological and microbiological diagnostic tools, the collection of detailed demographic, occupational and clinical characteristics to investigate risk factors for TBI, and the participation of non-medical healthcare workers to explore occupational risk of TB.

## Conclusion

Indonesia is a high incidence setting for TB, and nosocomial and occupational TB hinder TB elimination efforts. This study supports prioritisation of HCWs as a high-risk group for TB infection and disease, and the need for comprehensive prevention and control programs in Indonesia. These include annual staff TB screening, ensuring implementation of IPC procedures in facilities, TB infection and disease surveillance, access to preventive therapy and clear national guidelines. Further, it identifies characteristics of HCWs in Yogyakarta at higher risk, identifying certain groups who could be prioritised in screening programs if universal coverage of prevention and control measures cannot be achieved.
